# Phylogenetic analysis of the complete mitochondrial genome of *Orestias ascotanensis* (Cyprinodontiformes, Cyprinodontidae)

**DOI:** 10.1080/23802359.2019.1687033

**Published:** 2019-11-11

**Authors:** Fang Meng, Jian Chen, Zengliang Miao, Kehua Zhu, Youkun Huang, Qi Wang, Bingjian Liu, Yifan Liu

**Affiliations:** a National Engineering Research Center for Marine Aquaculture, Zhejiang Ocean University, Zhoushan, China;; b National Engineering Laboratory of Marine Germplasm Resources Exploration and Utilization, Marine Science and Technology College, Zhejiang Ocean University, Zhoushan, China

**Keywords:** *Orestias ascotanensis;* mitochondrial genome, evolutionary relationships

## Abstract

The complete mitochondrial genome of this species was first determined in this study, which is 16,617 bp in length, containing 13 protein-coding genes, 2 rRNA genes, 22 tRNA genes, a putative control region, and 1 origin of replication on the light-strand. The overall base composition includes C(27.11%), A(26.68%), T(29.15%), G(17.04%) and three degenerate bases are R, R and S. Moreover, the 13 PCGs encode 3800 amino acids in total, 12 of which use the initiation codon ATG except COI that uses GTG. Most of them have TAA as the stop codon, whereas ND5 ends with AGA, four protein-coding genes (ND1, ND2, ND3 and Cytb) ended with TAG, and two protein-coding genes (COII and ND4) ended with the incomplete stop codon represented as a single T. The phylogenetic tree based on the Neighbor Joining method was constructed to provide relationship within Cyprinodontidae, which could be a useful basis for management of this species.

*Orestias ascotanensis*, belongs to the family *Orestias*, Cyprinodontiformes, is a subtropical fish which distributed in the waters of the Ascotán Lake in Chile, South America, and inhabits the middle and bottom waters (Morales et al. 2011). Although two complete mitochondrial (mt) genomes belonging to *O. ascotanensis* have been determined, the *O. ascotanensis* mt genome sequence has not been reported yet.

Here, we sequenced and characterized the complete mt genome of *O. ascotanensis*. The specimen was collected from Ascotán Lake, Chile, South America (21°5′2″S, 70°7′0″W) and stored in a refrigerator of −80 °C in National Engineering Research Center for Marine Aquaculture (Accession number: OA180917). Total genomic DNA was extracted from muscle tissue of individual using the phenol–chloroform method (Barnett and Larson 2012). The calculation of base composition and phylogenetic construction was conducted by MEGA 6.0 software (Tamura et al. 2013). Similar to the typical mitogenome of vertebrates, the mitogenome of *O. ascotanensis* is a closed double-stranded circular molecule of 16,617 bp (GenBank accession No. MN514981), which contains 13 protein-coding genes, 2 ribosomal RNA genes, 22 tRNA genes, and 2 main non-coding regions (Boore 1999; Zhu, Gong, et al. 2018; Zhu, Lu, et al. 2018; Gong et al. 2017). The overall base composition is 26.68%, 29.15%, 27.11%, and 17.04% for A, T, C, and G, respectively. In addition, it also includes three degenerate bases are R, R and S. Most mitochondrial genes are encoded on H-strand except for ND6 and eight tRNA genes (tRNA-Gln, tRNA-Ala, tRNA-Asn, tRNA-Cys, tRNA-Tyr, tRNA-Ser, tRNA-Glu and tRNA-Pro), which are encoded on the L-strand. All of them use the initiation codon ATG except COI uses GTG, which is quite common in vertebrate mtDNA (Miya et al. 2001; Li et al. 2016; Jiang et al. 2018). Most of them have TAA as the stop codon, whereas ND5 ends with AGA, four protein-coding genes (ND1, ND2, ND3 and Cytb) ended with TAG and two protein-coding genes (COII and ND4) ended with an incomplete stop codon T (Ojala et al. 1981). The 12S rRNA and 16S rRNA are 943 and 1689 bp, which are both located in the typical positions between tRNA-Phe and tRNA-Leu, separated by tRNA-Val.

To explore the phylogenetic position of this *O. ascotanensis*, a phylogenetic tree was constructed based on the NJ analysis of 12 PCGs encoded by the heavy strand. The results of the present study suggest that *O. ascotanensis* has a closest relationship with *Poeciliopsis monacha*, highly supported by a bootstrap value of 100 (Figure 1), which are consistent with the results based on morphology and other molecular methods.

**Figure 1.**
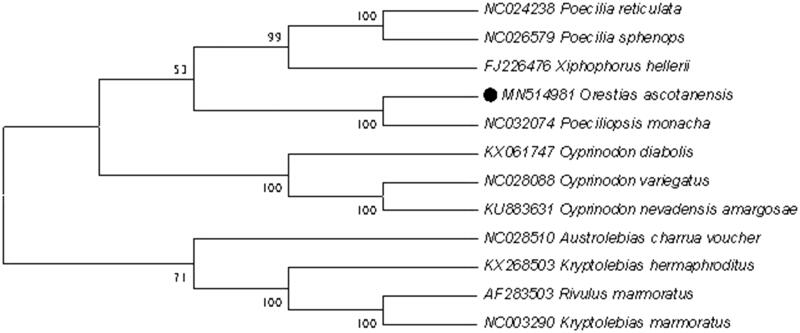
Neighbor joining (NJ) tree of 12 Cyprinodontiformes species based on 12 PCGs. The bootstrap values are based on 10,000 resamplings. The number at each node is the bootstrap probability. The number before the species name is the GenBank accession number. The genome sequence in this study is labeled with a black spots.
